# Influence of Bulk Nanobubbles Generated by Acoustic Cavitation on Powder Microstructure and Rehydration Characteristics of Spray-Dried Milk Protein Concentrate Powders

**DOI:** 10.3390/nano13061093

**Published:** 2023-03-17

**Authors:** Karthik Sajith Babu, Jayendra K. Amamcharla

**Affiliations:** Department of Animal Sciences and Industry, Food Science Institute, Kansas State University, Manhattan, KS 66506, USA

**Keywords:** milk protein concentrate, nanobubbles, acoustic cavitation, microstructure, rehydration behavior

## Abstract

Bulk nanobubbles (BNBs) have widespread applications in various fields of science due to numerous peculiar characteristics. Despite significant applications, only limited investigations are available on the application of BNBs in food processing. In the present study, a continuous acoustic cavitation technique was used to generate bulk nanobubbles (BNBs). The aim of this study was to evaluate the influence of BNB incorporation on the processability and spray drying of milk protein concentrate (MPC) dispersions. MPC powders were reconstituted to the desired total solids and incorporated with BNBs using acoustic cavitation as per the experimental design. The control MPC (C-MPC) and BNB-incorporated MPC (BNB-MPC) dispersions were analyzed for rheological, functional, and microstructural properties. The viscosity significantly decreased (*p* < 0.05) at all the amplitudes studied. The microscopic observations of BNB-MPC dispersions showed less aggregated microstructures and greater structural differences compared with C-MPC dispersions, therefore lowering the viscosity. The viscosity of BNB incorporated (90% amplitude) MPC dispersions at 19% total solids at a shear rate of 100 s^−1^ significantly decreased to 15.43 mPa·s (C-MPC: 201 mPa·s), a net decrease in viscosity by ~90% with the BNB treatment. The control and BNB incorporated MPC dispersions were spray-dried, and the resultant powders were characterized in terms of powder microstructure and rehydration characteristics. Focused beam reflectance measurement of the BNB-MPC powders indicated higher counts of fine particles (<10 μm) during dissolution, signifying that BNB-MPC powders exhibited better rehydration properties than the C-MPC powders. The enhanced powder rehydration with the BNB incorporation was attributed to the powder microstructure. Overall, reducing the viscosity of feed by BNB incorporation can enhance the performance of the evaporator. This study, therefore, recommends the possibility of using BNB treatment for more efficient drying while improving the functional properties of the resultant MPC powders.

## 1. Introduction

The milk protein concentrates (MPC) are manufactured from skim milk using ultrafiltration and diafiltration to selectively remove lactose and minerals. Subsequently, unit operations such as reverse-osmosis/evaporation and spray drying are employed to manufacture MPC in powder form. Simple and innovative strategies before spray drying are of critical consideration for the dairy industry producing MPCs. One of the major concerns for the manufacture of dairy powders is the energy-associated environmental footprint. There were findings that showed that the energy footprint of milk powders is seven times more than packaged milk; therefore, to meet the future need for a significant reduction in energy consumption, innovative technologies are needed [1,2]. This could be attributed to the high water content in milk and the energy-intensive thermal processing required for this water removal. Thus, ideally, for an energy-efficient process, minimizing thermal inputs is the key. Remarkably, Fox et al. [3] reported that an increase of just 2% dry matter could achieve 6% energy reduction. However, drying high solids is challenged by equipment performance constraints and product quality due to feed concentrate physical properties. Viscosity, in particular, is a significant concern while increasing dry matter content in the feed concentrate. Reducing viscosity also translates to reduced fouling of heat exchangers, improved heat transfer during evaporation, and a reduced amount of blocking in the spray drying nozzles. It is important for the dairy industry to adopt new technologies and strategies to reduce the viscosity of spray dryer feed even at higher total solids (TS); this can offer significant savings on the overall energy cost of powder production. Therefore, any innovative approaches for reducing the viscosity of high solids concentrate could be a promising step to higher energy savings. Many processing strategies were investigated to reduce the viscosity of the feed concentrates. This can be achieved either by altering the protein–protein interactions and/or by employing techniques such as acoustic cavitation [4], high-pressure homogenization [5], CO_2_ injection [6], etc. Recently, the key parameters and strategies to control the viscosity of dairy concentrates in the manufacturing of milk powders were reviewed [7].

The past two decades have seen substantial industrial and academic importance in investigations of the unique properties of ultrafine bubbles or nanobubbles (NBs). Ordinary bubbles have larger diameters, causing them to rise immediately to the surface of an aqueous solution, collapse or burst out of the solution, and disappear. However, NBs were demonstrated to remain in liquid for an extended period of time [8]. NBs are gas-filled cavities within liquids having diameters < 1 µm. Two types of NBs were studied: (i) surface nanobubbles, which form at solid–liquid interfaces, and (ii) bulk nanobubbles (BNBs), which exist in the bulk liquid. Unlike ordinary bubbles, NBs exhibit high stability, negative surface charge, and a large surface-to-volume ratio. In the present study, we explored the possibility of generating BNBs using a continuous acoustic cavitation technique. Very recently, the proof of the existence of BNBs generated using acoustic cavitation was given by [9]. Acoustic cavitation involves the generation, expansion, growth, and adiabatic collapse of microbubbles. The collapse and disappearance of such microbubbles give rise to the formation of BNBs. The effects of ultrasonic frequency and power on the formation of NBs were previously investigated [10]. The BNBs were created in their study by sonicating ultrapure water, and the concentration of BNBs are gaining popularity because they can enhance surface area, promote mass transfer, and change the physicochemical properties of a medium [11]. Lately, the experimental works on the existence of BNBs were debated by various research groups. One research group claimed to have presented unquestionable evidence for the existence of BNBs in pure water [9]. In contrast, another research group clearly disagreed and ruled out the possibility of the existence of BNBs in pure water [12,13]. Rak et al. [12] demonstrated that acoustic cavitation generates fine metal (titanium) nanoparticles from the disintegration of the surface of the ultrasonic probe, leading to the generation of nanoparticles that are, however, not BNBs. The existence and stability of NBs were verified and applied in a variety of science fields. Although theoretical research on BNBs is currently lacking, multiple applications of BNBs in diverse sectors, such as water treatment, surface cleaning, and food and agriculture applications, were previously reviewed [14,15,16]. Ultrasound found applications in various fields, such as mineral flotation [17], bioleaching [18], metal extraction [19], and crystallization [20]. The effect of ultrasonic treatment on dairy proteins was previously reviewed by Munir et al. [21]. The molecular space in any liquid medium is compressed and stretched as ultrasound travels through the liquid. Consequently, cavitation bubbles comprising of liquid vapors and dissolved gases are created when the minimum molecular distance necessary to keep the liquid intact is exceeded by the distance between the molecules of the liquid. With each cycle, the size of these bubbles grows until they reach a critical size during subsequent compression, at which point they implode [18]. While it was always anticipated that microbubbles will collapse and disappear, in this case, we anticipated that their absence caused previously undetectable nanobubbles to emerge. However, it is also plausible that acoustic cavitation can be used to produce these BNBs directly [9]. Several studies investigated the bulk and surface nanobubbles generated from ultrasonic irradiation; however, the applications of these nanobubbles on food protein systems are still very limited.

One of the key challenges encountered by the MPC manufacturers during the processing is the high viscosity after ultrafiltration and evaporation. Reducing viscosity and aiding an increase in the solid levels before spray drying can offer significant savings on the overall energy cost of powder production. On the other side, it is also a challenge for the end-user to incorporate the MPC powders in a formulation due to their poor rehydration properties, which are influenced by intrinsic powder properties, such as surface and bulk composition, rehydration conditions, storage, etc. [22]. Mechanical pre-treatments before spray drying were also shown to improve the solubility and other functional properties of MPC powders: nitrogen gas injection [23,24], extrusion porosification [25], and hydrodynamic cavitation [26]. This study aims to (i) characterize the BNBs generated by continuous acoustic cavitation in deionized water (DI) in terms of concentration and mean diameter; (ii) evaluate the effect of BNBs on MPC dispersions, and (iii) investigate the microstructure and rehydration characteristics of BNB-incorporated MPC powders.

## 2. Materials and Methods

### 2.1. Materials and Experimental Design

**Figure 1:**
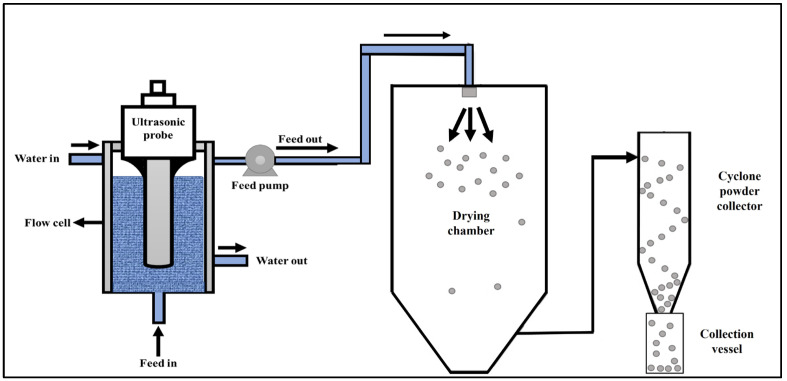
Schematic for the experimental setup for continuous bulk nanobubble incorporation during spray drying.

**Figure 2:**
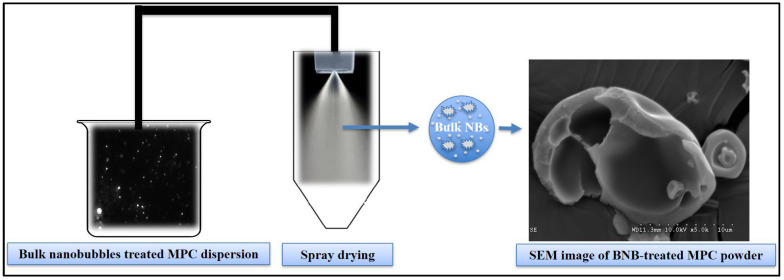
Schematic diagram of the proposed mechanism of bulk nanobubble incorporation during spray drying.

The effect of acoustic cavitation on BNBs were previously reviewed by Bu and Alheshibri, [27]. BNBs were generated by continuous acoustic cavitation using a 20 kHz probe-type processor (VCX 1500, Sonics & Materials, Newtown, CT, USA) capable of delivering up to 1.5 kW of power with a flow cell design, as shown in Figure 1. A new clean titanium probe of 1-inch diameter and 9-inch length was used to acoustically cavitate the bulk liquid flowing at a rate of 10 mL/min using a peristaltic pump (Watson Marlow, Wilmington, MA, USA). The amplitude during acoustic cavitation varied from 50% to 90%. The temperature of the sample throughout the BNB incorporation was controlled at 20 °C by using a recirculating cooler. Fresh MPC powder was supplied by a commercial dairy ingredient manufacturer within the USA. The powders were procured and immediately stored in refrigerated conditions until the planned experiments were undertaken. Three lots of MPC85 powders were obtained from a commercial manufacturer, and a randomized complete block design with TS (15 and 19%, *w*/*w*) and ultrasonic amplitude (0, 50, 75, and 90%) as independent factors were studied. The average protein, moisture, fat, lactose, and ash content of the MPC85 used for this study was 86.05% (*w*/*w*), 5.11% (*w*/*w*), 0.93% (*w*/*w*), 4.81% (*w*/*w*), and 6.65% (*w*/*w*), respectively. Briefly, MPC85 powders were reconstituted to 15 and 19% TS in DI water by gradual addition of powder using a 4-bladed overhead stirrer (Caframo, Georgian Bluffs, ON, Canada) with a temperature-controlled water bath (Fisher Scientific, Pittsburgh, PA, USA) maintaining the powder dissolution temperature at 40.0 ± 0.1 °C. The overhead stirrer speed was set at 900 rpm during powder addition. Subsequently, the stirrer speed was reduced to 400 rpm. The prepared MPC feed samples were left overnight in a refrigerator to ensure complete rehydration of the powder. Samples were equilibrated to 20 °C in a water bath following overnight storage, and BNBs were incorporated using the continuous acoustic cavitation system as per the experimental design. Control MPC (C-MPC; pumped through the BNB generation system without BNB treatment) and BNB-incorporated MPC (BNB-MPC; BNB incorporated) dispersions were compared and evaluated for physical, rheological, morphological, and functional properties. In addition, selected combinations (19% TS; amplitude 50 and 90%) were spray-dried using the spray dryer (YC-015; Shanghai Pilotech Instrument & Equipment Co., Ltd., Shanghai, China) to characterize and compare the resultant BNB-MPC powder with the C-MPC powder. The inlet temperature was set at 180 °C, and the outlet temperature ranged between 60 and 65 °C. The spray pressure was maintained at 206.84 kPa, and the relative humidity of the room was periodically recorded using a digital humidity meter (Traceable Humidity Meter, Fisher Scientific). Figure 2 illustrates the potential mechanism of BNBs. Conventional spray drying of MPC resulted in particles with a hollow interior surrounded by a highly compacted shell. In contrast, with the BNB incorporation, at the early stage of air drying, it is believed that the BNBs escape and burst within the atomized droplet, resulting in porous structured MPC powder particles. The spray-dried C- and BNB-MPC powders were collected and sealed in Whirl-Pak bags (Nasco) for further analysis.

### 2.2. Characterization of Bulk Nanobubbles

The control and BNB-incorporated DI water were characterized in terms of size and concentration using the Malvern NanoSight LM10 (Malvern Instruments Ltd., Malvern, UK) nanoparticle tracking analysis (NTA) system. NTA a firmly established method that gives real-time visualization, size, and concentration of nanoparticles or NBs in the bulk liquid. All the measurements were performed at 25 °C. The dispersed light from numerous separate nanoscale particles were monitored and the instrument was equipped with a charged-coupled device camera. As a result, the size and relative scattering intensity of each nanoscale particle that scattered light in the water samples were calculated. For each sample, 5 × 60 s videos were recorded, and the parameters used were camera level = 10, threshold = 2. Particles detected by this technique ranged from 10 to 1000 nm in diameter. NTA could not differentiate between the nanoparticles and the BNBs. In addition, the non-existence/traces of titanium nanoparticles were also determined using inductively coupled plasma mass spectrometry. The pure water was incorporated with BNBs and then transferred to the Perkin Elmer NexIon 350 inductive coupled plasma-mass spectrometry for analyzing titanium concentration in bulk NB suspensions. These measurement techniques and their protocols are discussed in more detail elsewhere [9,28].

### 2.3. Rheological Measurements of C- and BNB-MPC Dispersions

The C- and BNB-MPC liquid dispersions were characterized in terms of their rheological properties immediately after incorporation of bubbles. To further understand whether the observed rheological changes were temporary or not, the C- and BNB-MPC liquid dispersions were frozen and analyzed for rheology after thawing. The frozen samples of the C- and BNB-MPC dispersions were thawed in a refrigerator at 4 °C for a day, followed by further thawing in a water bath at 45 °C for 30 min, and were tested for rheological properties. The continuous rotational flow measurement was carried out using a stress–strain-controlled rheometer (MCR-92 Anton Paar) at a varied shear rate from 0.01 s^−1^ to 100 s^−1^ at 20 °C, and the data obtained were also fitted to the power-law model.

### 2.4. Microstructure of C- and BNB-MPC Dispersions

The microstructure of C-MPC and BNB-MPC dispersions were studied using confocal laser scanning microscopy (CLSM), following the method described by Gandhi et al. [29]. The microstructures were visually compared, and representative areas were chosen for display in the manuscript for the selected C-MPC and BNB-MPC dispersions (15 and 19% TS; amplitude 0 and 90%). The microstructure was studied to further support the viscosity effects on BNB treatment. Samples were prepared for CLSM by diluting 1:100 (*v*/*v*) using DI water before measurements. Proteins were stained using the Fast green FCF (Sigma-Aldrich, St. Louis, MO, USA) stain. Stock solution of Fast green (5 mg dye in 5 mL water) was applied to the sample for 5–10 min. The stained samples were analyzed in LSM 5 Pa (Zeiss, Thornwood, NY, USA) and were analyzed using the software (LSM-% PASCAL Version 3.2sp2). Three-dimensional images were obtained by scanning the sample across a defined section along the *z*-axis. Transmission electron microscopy (TEM) was also used to image C- and BNB-MPC dispersions in order to characterize the microstructure. Samples were analyzed for morphology using negative-staining technique. One drop of the diluted sample was mounted on Formvar/carbon-coated 200-mesh copper grids (Electron Microscopy Sciences, Fort Washington, PA, USA) for 2 min. Subsequently, combined with a drop of 2% uranyl acetate and left for 30 s and the excess liquid was wiped with a filter paper and was examined using a CM 100 TEM (FEI Company, Hillsboro, OR, USA) operating at 100 kV, and images captured with a digital camera (model C8484, Hamamatsu, Bridgewater, NJ, USA).

### 2.5. Particle Size Distribution of C- and BNB-MPC Dispersions

The average particle size of C- and BNB-MPC dispersions were measured at 20 °C by dynamic laser light scattering using a particle size analyzer (DelsaMax PRO; Beckman Coulter, Brea, CA, USA) at a scattering angle of 171°. The C- and BNB-MPC dispersions were prepared for particle size analysis by diluting 1:100 (*v*/*v*) using DI water before measurements.

### 2.6. Microstructure of Spray-Dried C- and BNB-MPC Powder

The microstructure of C- and BNB-MPC powders were examined using a scanning electron micrography (SEM) according to the method described by Bouvier et al. [25]. The C- and BNB-MPC powders were chopped at room temperature using an injector blade (71990, Electron Microscopy Sciences, Hatfield, PA, USA). After chopping, the C- and BNB-MPC powders were directly mounted onto a carbon double-sided adhesive tape on microscopy stubs and sputter-coated with palladium using a Denton Vacuum Desk II sputter coater (Denton Vacuum, Moorestown, NJ, USA) for 15 min to avoid the charge buildup under the electron beam. The imaging for obtaining the cross-section of C- and BNB-MPC powders samples were performed using the S-3500N (Hitachi Science Systems Ltd., Tokyo, Japan) version 10-16-2266 (PC) 10-04 (SEM) and was examined by a secondary electron detector operating at 10 kV and at magnification range of ×500 to ×4000 was obtained. The images were produced at Nanotechnology Innovation Center of Kansas State University (Manhattan, KS, USA).

### 2.7. Rehydration Characteristics of Spray-Dried C- and BNB-MPC Powders

The rehydration behavior of the C- and BNB-MPC powder were studied using three techniques: the solubility of the powders was estimated based on the TS in the supernatant obtained by centrifugation at 700× *g* for 10 min at 25 °C, as described by [30]. Then, the amount of soluble material in the MPC was calculated. Additionally, the dissolution characteristics of the C- and BNB-MPC powders were evaluated using the focused beam reflectance measurement (FBRM) [31] and electrical resistance tomography (ERT) [32] methods.

### 2.8. Statistical Analysis

Measurements of C- and BNB-MPC dispersion and powder characteristics were performed in triplicate, with results presented as mean ± standard deviation. Data were analyzed as a completely randomized design with TS (15 and 19% TS, *w*/*w*) and amplitude (0, 50, 75, and 90%) as independent factors for C- and BNB-MPC dispersions and 19% TS with an amplitude of 50 and 90% for C- and BNB-MPC powders. Analysis of variance was carried out using the statistical software SAS (PROC GLIMMIX, Version 9.4; SAS^®^ Institute, Inc., Cary, NC, USA) statistical analysis package. The level of significance was set at *p* < 0.05.

## 3. Results and Discussion

### 3.1. Characterization of Bulk Nanobubbles

**Figure 3:**
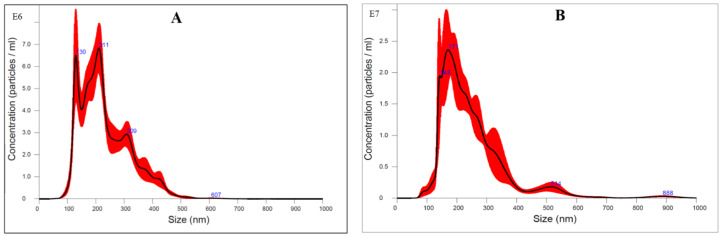
Nanoparticle tracking analysis results shown as a smoothed histogram presenting average concentration and size of control (**A**) and bulk nanobubble incorporated (**B**) in deionized water.

The control and the BNB-incorporated DI water was characterized in terms of concentration and mean diameter using the nanoparticle tracking system. BNB-incorporated DI water (90% amplitude) had a mean particle size of 235.5 ± 102.3 nm. The particle size distribution of the BNBs was D10, 134.3; D50, 209.2; D90, 369.6 nm. The concentration was found to be 2.41 × 10^8^ and 1.19 × 10^9^ particles/mL for control and BNB-incorporated (90% amplitude) DI water, respectively. In other words, the concentration of BNB-incorporated DI water was significantly higher compared to the DI water (~1 billion NBs/mL). One of the common methods that was reported to produce BNBs was based on acoustic cavitation [9,28]. NTA and inductively coupled plasma mass spectrometry were used to provide evidence of the existence of BNBs generated by acoustic and to demonstrate that the nano-entities observed in DI water were indeed gas-filled domains (BNBs), and to test the efficiency of the continuous acoustic cavitation system to generate BNBs efficiently. Figure 3 shows the NTA results obtained from control and BNB-incorporated DI water. The NTA system showed a significant increase in particle concentration upon the BNB treatment, suggesting that the acoustic cavitation was efficient in generating BNBs. Previously, Yasuda et al. [10] generated BNBs from ultrasonic irradiation (frequency of 22 kHz) and noted a BNB size of 50–220 nm. Likewise, Jadhav and Barigou [9] and Nirmalkar et al. [28] also used acoustic cavitation and generated BNBs that were stable in pure water. Over the last decade, researchers debated whether the nano entities created were BNBs or other nanoparticles [33]. Notably, BNB-incorporated DI water samples had the presence of titanium in only a trace amount (0.00017 μg/mL), implying the presence of titanium contamination could not be blamed for the detected nano-entities using NTA, which agrees with the results reported by another research group [9,34]. This observation further confirmed the presence of BNBs generated via acoustic cavitation. In contrast, some researchers reported that the presence of nanoparticles were misinterpreted as BNBs [13]. Nevertheless, there is no definite consensus in the literature yet as to why the BNBs have incredible longevity, and BNB stability remains a mystery to date.

### 3.2. Viscosity Measurements

**Figure 4:**
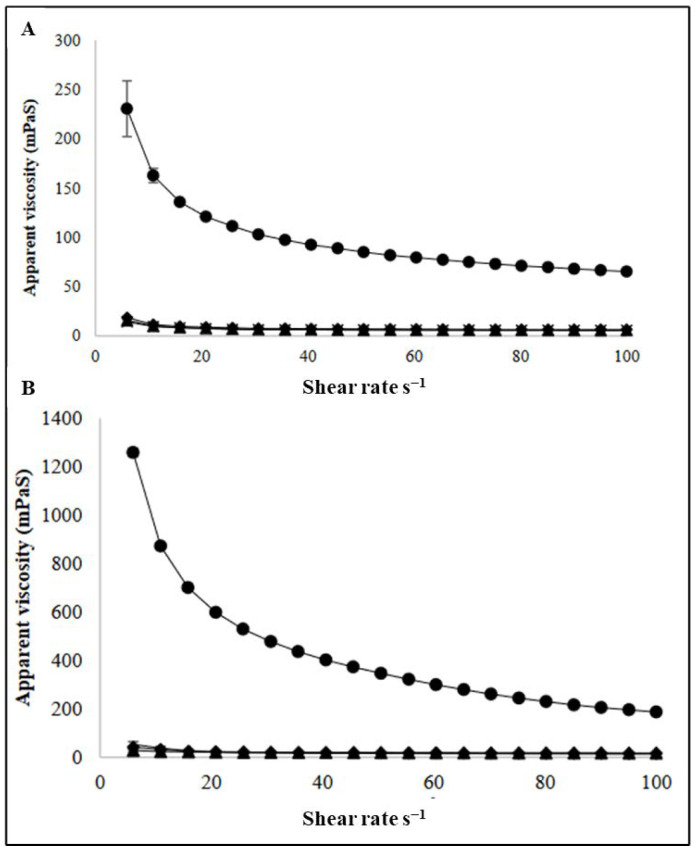
Apparent viscosity versus shear rate plot of control (●) and bulk nanobubble-incorporated milk protein concentrate dispersions (MPC): 15% (**A**) and 19% (**B**) total solids MPC dispersions at amplitude of 50 (×), 75 (♦), and 90% (▲), respectively. Values are the means of data from three lots of MPC powders, with the standard deviation indicated by error bars. Measurements were carried out at 20 °C.

The general flow characterization of the C- and BNB-MPC dispersions are shown in Figure 4. All samples exhibited a non-Newtonian shear thinning behavior. Compared to the C-MPC, the viscosity significantly decreased (*p* < 0.05) at all the amplitudes (50, 75, and 90%) studied. However, it was observed that with the increase in amplitude, the viscosity of MPC dispersions were not significantly (*p* > 0.05) different from each other. The apparent viscosity of the C-MPC was 68.90 and 201.57 mPa·s at 100 s^−1^ for 15 and 19% TS, respectively. The viscosity significantly (*p* < 0.05) decreased to 5.55 and 15.43 mPa·s at 100 s^−1^ for 15 and 19% TS, respectively, after the BNB incorporation (90% amplitude). A similar reduction in apparent viscosity values were observed after the treatment at 50 and 75% amplitude. This represents a net decrease of ~90% viscosity for BNB-MPC compared to the C-MPC. The observed differences in viscosity could be directly related to particle size and microstructure. A similar trend was reported in a study conducted by Li et al. [26] using hydrodynamic cavitation, with the decrease in viscosity in MPC retentates at cavitation frequencies of 25 and 50 Hz. Analysis of the viscosity profiles with the power-law model also highlighted the differences in the flow behavior index, *n*, and the consistency coefficient, κ, for the C- and BNB-MPC dispersions. At 19% TS, the *n* and κ values were 0.44 and 2.90 Pa Sn for C-MPC, whereas it was 0.74 and 0.06 Pa Sn for the BNB-MPC (90% amplitude) dispersion. Likewise, our previous research on MPC dispersions and Greek-style yogurt also demonstrated the viscosity-lowering effect of BNBs [35,36]. Similar results using ultrasound treatment were obtained by the work of Deshpande and Walsh [37] and Song et al. [38] in reconstituted MPC (30–44% TS) and skim milk powder (46–64% TS) and micellar casein concentrate, respectively. The results of the present study were also supported by Zisu et al. [4], who found a significant change in the rheological properties by acoustic cavitation of concentrated skim milk. They reported a reduction in viscosity of ~10%. Remarkably, the effect of acoustic cavitation was more pronounced for age-thickened samples, for which a reduction (~17%) was found. Chen et al. [39] hypothesized that during the acoustic cavitation process, bubble implosion produced shock waves, which led to viscous dissipative eddies that developed shear stresses in the medium, therefore lowering the viscosity. Additionally, when BNBs are introduced into the MPC systems, they function as a buffer between milk protein particles, thereby preventing the aggregation of the proteins. Previously, Yanjun et al. [40] studied the influence of ultrasound on MPC systems and found that sonication broke apart large aggregates leading to a decrease in particle size and a lower viscosity. Amamcharla et al. [41] reported that introducing bulk MNBs with an average diameter of 100 nm to 30 µm reduced viscosity in liquid dairy products. The viscosity reduction upon BNB treatment in apple juice concentrate and canola oil was demonstrated by Phan et al. [42]. Pathania et al. [43] demonstrated that hydrodynamic cavitation was more effective in rapidly rehydrating MPC powders in comparison with conventional high-shear treatment.

Furthermore, the freeze–thawed C- and BNB-MPC dispersions exhibited similar behavior to that of the samples tested immediately after the BNB incorporation, and a decreased viscosity was demonstrated at both total solids studied (Table 1 and Table 2). Researchers previously reported that when BNBs made in pure water were frozen and then thawed, they appeared to vanish, and the various causes of this disappearance are discussed elsewhere [9]. Interestingly, Jadhav and Barigou [44] observed that nano-entities floating in water disintegrated into fine bubbles after being frozen and thawed, which tended to re-cluster and restore their initial stable size fast when shaken vigorously or gradually if held undisturbed for days. For concentrated milk protein systems, it is well established that the presence of aggregates can result in a marked increase in viscosity [45]. Thus, based on the current findings, it was implied that the BNB incorporation using the acoustic cavitation-assisted decrease in viscosity was due to the disruption of aggregates. Overall, BNB treatment can be a more efficient processing approach than other conventional ones and can be envisaged as a novel “green” approach.

### 3.3. Microstructure of C- and BNB-MPC Dispersions

**Figure 5:**
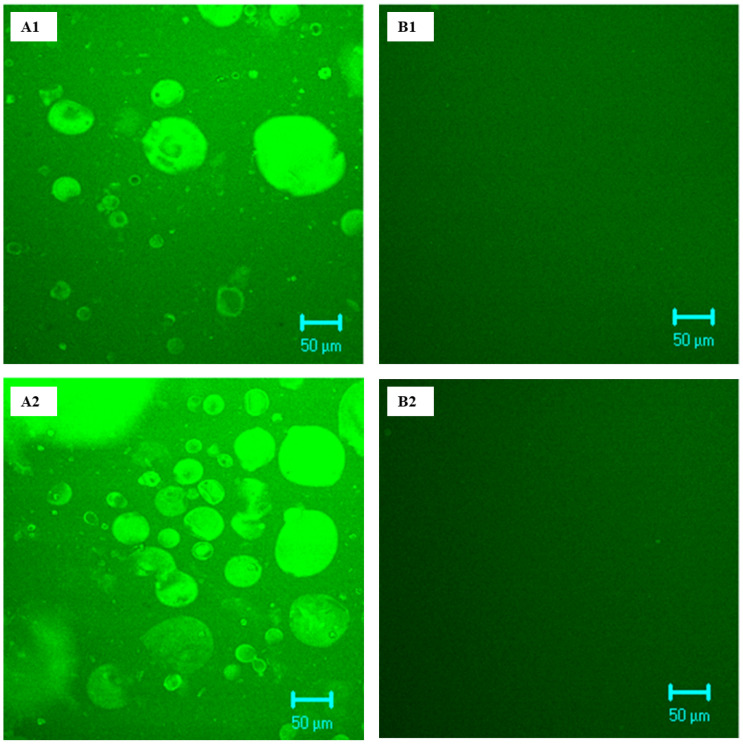
Representative confocal laser scanning microscopy images of milk protein concentrate dispersions: control at 15% (**A1**); bulk nanobubble-incorporated (90% amplitude) at 15% total solids; (**B1**); control at 19% total solids (**A2**); bulk nanobubble-incorporated (90% amplitude) at 19% total solids (**B2**).

**Figure 6:**
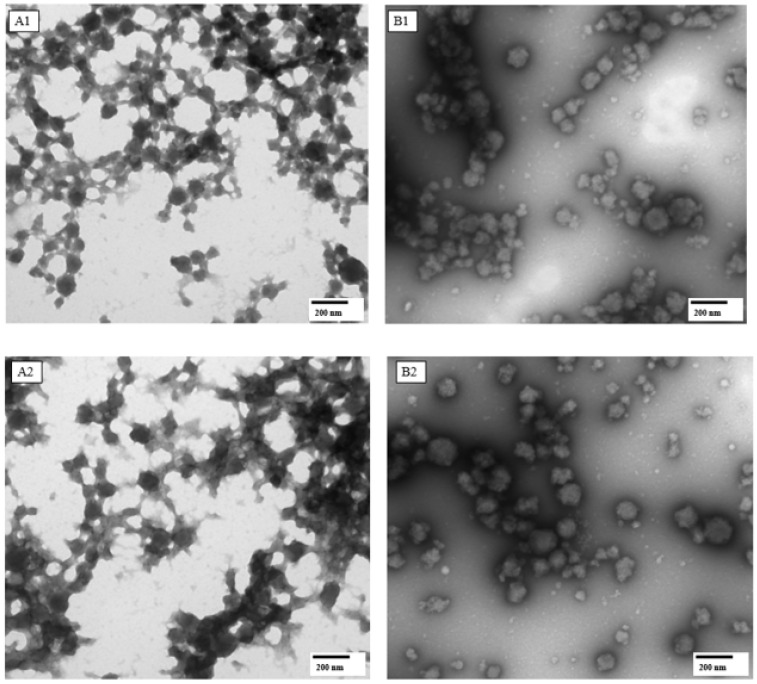
Representative transmission electron microscopy (TEM) images of milk protein concentrate dispersions: control at 15% (**A1**); bulk nanobubble-incorporated (90% amplitude) at 15% total solids; (**B1**); control at 19% total solids (**A2**); bulk nanobubble-incorporated (90% amplitude) at 19% total solids (**B2**). TEM magnification and scale: 34,000× and 200 nm, respectively.

The microstructure of C- and BNB-MPC dispersions (BNB-incorporated powders at 90% amplitude) is shown in Figure 5 and Figure 6. The rheological characteristics of dispersions incorporated with BNBs clearly suggested that the structural configurations changed. CLSM and TEM were used to analyze the microstructures of C-and BNB-MPC dispersions. The CLSM revealed noticeable microstructural changes upon the BNB treatment. The TEM micrographs of C-MPC dispersions revealed a dense cross-linked network structural design. In contrast, a less dense structure was seen in the BNB-MPC dispersion, along with a thinner protein network. This can be attributed to the physical shear caused by the BNB incorporation, which resulted in the protein aggregates being effectively redistributed, thus decreasing the particle size. However, the network structure appeared more homogenous and with a higher degree of branching for the C-MPC when the dispersions were not exposed to BNBs, which could explain the higher viscosity observed in the C-MPC dispersions. It was reported that the shear force and turbulence generated by acoustic cavitation could cause protein rearrangements, and the particle size of the complexes was significantly reduced [45,46,47]. The BNB-MPC dispersions showed a significant reduction in large aggregate formations. The structural disintegration of protein aggregates caused by bubble implosion during the hydrodynamic cavitation process might explain this. Previously, Babu et al. [36] reported visible microstructural changes in Greek-style yogurts upon NB treatment. Although TEM revealed a more scattered structure when the MPC system was incorporated with BNB, additional research is needed to determine if this is a transitory phenomenon or whether it results in a permanent viscosity decrease. Overall, the impact of BNB on the rheological characteristics of C- and BNB-MPC dispersions was clearly characterized based on structural analysis.

### 3.4. Particle Size Analysis

The results of particle size analysis of C- and BNB-MPC dispersions are given in Table 3. Analysis of the particle size distribution showed the presence of relatively large particles in the C-MPC, which could be attributed to the presence of larger protein aggregates. The cavitation treatments at all amplitudes introduced a significant decrease in mean particle size for both 15 and 19% TS. BNB treatment indicated an absence of insoluble particles in a size range >10 μm, playing a key role in controlling viscosity. Based on the analysis of particle size distribution, the viscosity reduction was suggested to be associated with a decrease in the presence of large particles, possibly due to the disruption of aggregates. These findings were also supported by CLSM and TEM images (Figure 5 and Figure 6). Yanjun et al. [40] showed that the particle size of MPC dispersions was reduced by acoustic cavitation because of the disruption of aggregated protein network (20 kHz; amplitude, 50%). Gordon and Pilosof [48] observed significant reductions in whey protein isolate particle size from 0.73 µm in control samples to 0.35, 0.25, 0.26, 0.20 µm after acoustic cavitation for 2, 5, 10, and 20 min at 20% (20 kHz). Furthermore, the cavitation was found to force physical effects such as micro-streaming and hydrodynamic shock waves due to cavitation bubbles, breaking up the molecular structure of the proteins, and thereby reducing size [49]. Similarly, Zisu et al. [50] used continuous acoustic cavitation (20 kHz; flowrate, 0.3 L/min; amplitude at 30 and 60%) in whey protein and milk protein retentate and noted that force generated through acoustic cavitation resulted in a decrease in particle size of aggregates.

### 3.5. Microstructure of Spray-Dried C- and BNB-MPC Powders

**Figure 7:**
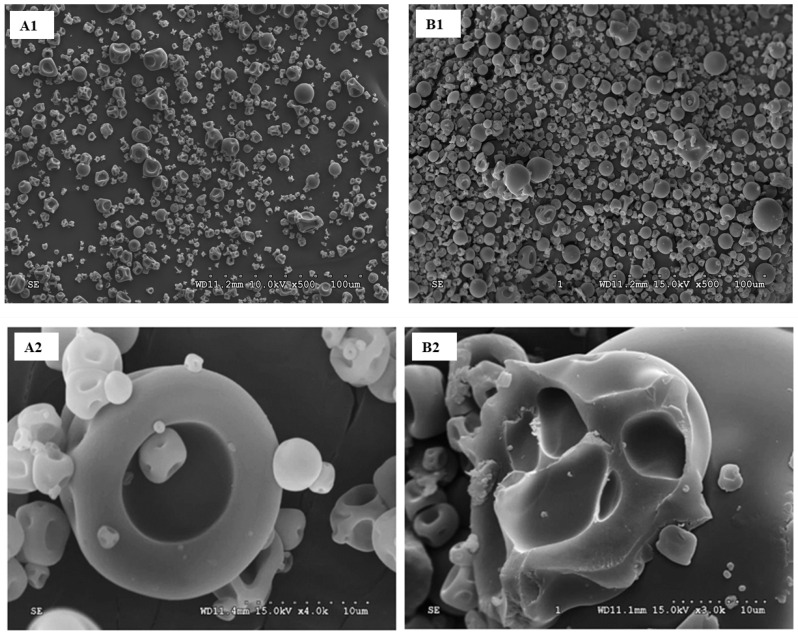
Typical scanning electron microscopy cross-sectional images of chopped spray-dried milk protein concentrate (MPC) powder particle: (**A1**,**A2**) control and (**B1**,**B2**) bulk nanobubble-incorporated (90% amplitude) MPC powders (1 and 2 is lower and higher magnifications, respectively).

The SEM analysis provided an overview of the shape and size of the C-and BNB-MPC powder particles. Noticeable changes in the microstructure of C-and BNB-MPC powders (19% TS and BNB-incorporated powders at 90% amplitude) were revealed from the SEM analysis (Figure 7). The C-MPC powder particle displayed a hollow interior surrounded by a highly compacted shell, whereas the BNB incorporation resulted in particles with numerous holes of different sizes throughout the powder particle matrix. Indeed, the particles associated with the BNB-incorporated powders with these porous structures were proposed to improve the rehydration behavior of the BNB-MPC powders. McSweeney et al. [23] and Bouvier et al. [25] used N_2_ and CO_2_/extrusion porosification, respectively, before spray drying, to produce MPC powders with similar particle structures. Likewise, the BNB incorporation also resulted in MPC powders with these porous structures and the presence of large air voids between the particles. Furthermore, this was also anticipated to facilitate increased water transfer, while also increasing the physical space between casein micelles and was believed to cause a faster structural collapse of the MPC powder particles when added to water [23].

### 3.6. Rehydration Characterization of Spray-Dried C- and BNB-MPC Powders

**Figure 8:**
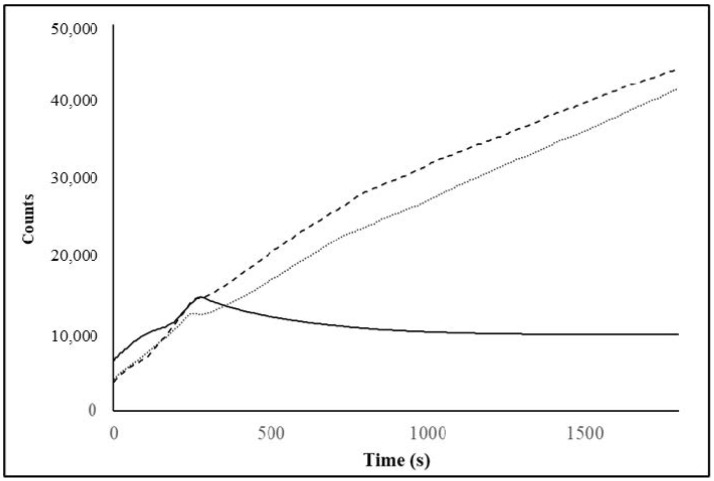
Changes in fine (<10 μm) counts obtained from data collected using the focused beam reflectance measurement for the spray-dried milk protein concentrate (MPC) powders: (-) control, (..) bulk nanobubble-incorporated (50% amplitude), and (--) bulk nanobubble-incorporated (90% amplitude) MPC powders at a dissolution temperature of 20 °C.

**Figure 9:**
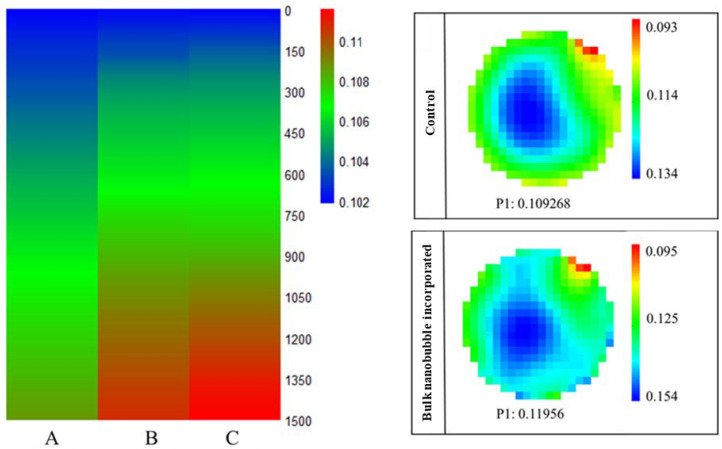
Overall mean conductivity results obtained from the electrical resistance tomography system (A) control, (B) bulk nanobubble-incorporated (50% amplitude), and (C) bulk nanobubble-incorporated (90% amplitude) milk protein concentrate powders at a dissolution temperature of 20 °C. Representative tomogram images obtained from the electrical resistance tomography system at the end of the dissolution time.

When comparing the rehydration behavior of the two powders, the BNB-MPC powders showed a much higher solubility index than C-MPC powders. The solubility index of the C-and BNB-MPC powders were 83 ± 0.69, 91.99 ± 0.68 (50% amplitude), and 94.57 ± 0.33% (90% amplitude), respectively. Very recently, McSweeney et al. [18] observed an improved dissolution of N_2_-injected MPC powders (control: 83.6% and N_2_-treated powders: 96.2%). The more porous structure of BNB-MPC powder particles and the presence of large air voids between these powder particles aided in the enhanced water transfer while also improving the physical space between casein micelles and reducing protein–protein interactions [18]. Similarly, Marella et al. [6] observed an improved dissolution of CO_2_-injected and stored (0–180 day) MPC powders. The change in solubility between the control and CO_2_-treated samples suggests that calcium and other minerals were involved in MPC solubility behavior. In contrast, Li et al. [26] used hydrodynamic cavitation and observed that the solubility values did not vary much for the MPC powders (dissolution at room temperature control: control—88.63% and treated-rotor speed at 50 Hz: 89.22%). Previously, Yanjun et al. [40] observed an increased solubility (35.78% to 88.30%) of acoustically pre-treated MPC powders after 5 min of ultrasound treatment. The rehydration properties of the BNB-incorporated MPC powders were examined and compared with those of C-MPC powders using the FBRM technique. The FBRM tracked the number of fine particles (<10 µm) as a function of dissolution time (Figure 8). The results showed that for the BNB-MPC powders, the large particles disintegrated into fine particles at a faster rate compared to C-MPC powders, suggesting water was able to penetrate the particles faster to dissolve quickly. The rate of water transfer during rehydration was supported by the presence of these large air voids and pores throughout the particle matrix. Bouvier et al. [25] reported similar solubility improvements using extrusion-porosification. Gandhi et al. [29] established that the rate of increase in fine counts were higher for well-soluble MPC powders. ERT was used to determine the rehydration characteristics. Figure 9 shows the overall mean conductivity of the C-and BNB-MPC powders using the circular configuration. At the end of the 15 min dissolution time, the overall mean conductivity from the circular configuration was found to be 0.108 ± 0.004 mS/cm for C-MPC powders, which was significantly (*p* < 0.05) different from the BNB-MPC powders. The BNB-incorporated MPC powders at amplitude 50 and 90% had overall mean conductivity of 0.110 ± 0.025 mS/cm and 0.111 ± 0.004 mS/cm (90%), respectively. Furthermore, tomograms were stacked to form a 3D image and the differences were evident form the tomograms. At the end of the 15 min dissolution time, dissolution characteristics for MPC powders were evident and are presented in Figure 9. Previously, Mimouni et al. [51] noted that the minerals were freely released during MPC powder rehydration, but the rate-limiting stage was the protein dispersion. Udabage et al. [52] investigated the application of the static high-pressure treatment on MPCs and reported an increase in solubility for the treated samples compared to the control MPC powders. As the dissolution time increased, more minerals were being released, and an increase in conductivity was visible. The sensing plane P1 had an overall mean conductivity of 0.109 mS/cm for C-MPC powders; however, for BNB-MPC powders, sensing plane P1 had a higher overall mean conductivity of 0.119 mS/cm. This result was likely related to the microstructure of the BNB-MPC powders. Additionally, BNB treatment was also reported to improve ultrafiltration membrane performance and flow properties of spray-dried milk protein concentrate powders [53,54], thereby making BNB treatment very appealing to various stages of MPC production.

## 4. Conclusions

In the present study, we investigated a method for generating BNB using the acoustic cavitation technique. The NTA measurements revealed that bubbles produced in DI water had a mean diameter of 235.5 ± 102.3 nm. The BNB treatment was used as a mechanical pre-treatment to improve the rheological characteristics of MPC dispersions. The BNB treatment of MPC dispersions lowered the viscosity by ~90% at 100 s^−1^ shear rate. Lowering the viscosity of the feed offered the possibility of drying MPC retentates at significantly higher solid concentrations and, thus, more efficient spray drying. Moreover, viscosity had a direct influence on the size of the droplets formed during atomization, reduced viscosity resulted in powder with improved powder properties. Being able to reduce the viscosity of the MPC retentates also results in a reduction in both capital and operational costs. Based on the viscosity data, a 50% amplitude was sufficient to significantly improve the processability of the MPC dispersions; however, during scale-up to meet the spray dryer requirements, this could be further optimized. Notable differences in the microstructure were also observed between the C-MPC and BNB-MPC dispersions subjected to the BNB treatment. Furthermore, the use of BNB treatment offered significant improvements to the rehydration behavior of the MPC powder particles. Thus, this study illustrated how BNB treatment can be employed in MPC processing. Additionally, BNB treatment is an eco-friendly, scalable, and robust process technology.

## Figures and Tables

**Table 1:** Rheological characterization of control and bulk nanobubble-incorporated milk protein concentrate dispersions (Total solids: 15%).

Amplitude (%)	Apparent Viscosity at 100 s^−1^ (mPaS)	Consistency Coefficient (PaS *^n^*)	Flow Behavior Index (η)	Apparent Viscosity at 100 s^−1^ after Thawing (mPaS)
Control	68.90 ± 2.67 ^a^	0.84 ± 0.04 ^a^	0.42 ± 0.06 ^a^	91.71 ± 3.02 ^a^
50	6.72 ± 1.21 ^b^	0.05 ± 0.03 ^b^	0.54 ± 0.07 ^b^	14.20 ± 1.25 ^b^
75	6.09 ± 0.21 ^b^	0.05 ± 0.02 ^b^	0.51 ± 0.08 ^b^	13.48 ± 0.02 ^b^
90	5.55 ± 0.78 ^b^	0.03 ± 0.01 ^b^	0.62 ± 0.06 ^b^	13.13 ± 1.11 ^b^

^a,b^ Mean within a column with different superscript differ (*p* < 0.05), Values are means ±SD, *n* = 3, measurements were carried out at 20 °C.

**Table 2:** Rheological characterization of control and bulk nanobubble-incorporated milk protein concentrate dispersions (Total solids: 19%).

Amplitude (%)	Apparent Viscosity at 100 s^−1^ (mPaS)	Consistency Coefficient (K) (PaS *^n^*)	Flow Behavior Index (η)	Apparent Viscosity at 100 s^−1^ after Thawing (mPaS)
Control	201 ± 13 ^a^	2.90 ± 1.23 ^a^	0.44 ± 0.08 ^a^	222 ± 10 ^a^
50	17.56 ± 0.48 ^b^	0.10 ± 0.03 ^b^	0.57 ± 0.03 ^a^	35.52 ± 1.55 ^b^
75	16.86 ± 0.83 ^b^	0.09 ± 0.03 ^b^	0.58 ± 0.03 ^a^	33.51 ± 2.01 ^b^
90	15.43 ± 0.52 ^b^	0.06 ± 0.01 ^b^	0.74 ± 0.05 ^a^	31.19 ± 0.59 ^b^

^a,b^ Mean within a column with different superscript differ (*p* < 0.05), Values are means ±SD, *n* = 3, measurements were carried out at 20 °C.

**Table 3:** Particle size and pH values of control and bulk nanobubble-incorporated milk protein concentrate dispersions.

Total Solids (%)	Amplitude (%)	Mean Diameter (nm)	pH
15	Control	214.25 ± 5.78 ^a^	6.72 ± 0.01 ^a^
	50	165.13 ± 2.12 ^b^	6.71 ± 0.01 ^a^
	75	161.81 ± 7.08 ^b^	6.71 ± 0.01 ^a^
	90	156.76 ± 7.29 ^b^	6.67 ± 0.01 ^b^
19	Control	304.74 ± 6.08 ^a^	6.73 ± 0.01 ^a^
	50	170.61 ± 4.57 ^b^	6.72 ± 0.02 ^a^
	75	163.69 ± 8.55 ^b^	6.73 ± 0.02 ^a^
	90	159.65 ± 8.39 ^b^	6.66 ± 0.01 ^b^

^a,b^ Mean within a column with different superscript differ (*p* < 0.05), Values are means ± SD, *n* = 3. The comparison was made within each group of total solids studied.

## Data Availability

Not applicable.
